# Height outcomes in children with growth hormone deficiency and idiopathic short stature treated concomitantly with growth hormone and aromatase inhibitor therapy: data from the ANSWER program

**DOI:** 10.1186/s13633-020-00089-z

**Published:** 2020-10-06

**Authors:** Bradley S. Miller, Judith Ross, Vlady Ostrow

**Affiliations:** 1 Department of Pediatrics, University of Minnesota, Minneapolis, MN USA; 2 Department of Pediatrics, Nemours duPont Hospital for Children, Thomas Jefferson University, Philadelphia, PA USA; 3 Novo Nordisk Inc, Plainsboro, NJ USA

**Keywords:** Human growth hormone, Aromatase inhibitors, Androgens, Growth disorders, Registries, Growth plate, Anastrozole, Letrozole, Adolescent

## Abstract

**Background:**

Treatment of children with growth hormone deficiency (GHD) or idiopathic short stature (ISS) using GH is only effective for bone growth prior to epiphyseal fusion. Aromatase inhibitor therapy (AIT) blocks estrogen production, thereby delaying epiphyseal fusion. The current study analyzed baseline characteristics and longitudinal data of male patients with GHD or ISS who were treated with GH and concomitant AIT.

**Methods:**

Data were obtained from the observational American Norditropin® Studies: Web-Enabled Research (ANSWER) Program, which collected efficacy and safety data of patients treated with Norditropin®. A longitudinal cohort approach compared patient characteristics, including chronologic age, bone age, and height standard deviation score (HSDS), in GH-treated males before and after AIT initiation.

**Results:**

A total of 142 GH-naïve patients with GHD (*n* = 115) or ISS (*n* = 27) with mean (± SD) baseline chronological ages of 12.10 ± 3.00 and 10.76 ± 3.07 years, respectively, were analyzed. The majority were classified at advanced Tanner stages II to V. Patients with GHD had mean HSDS of − 1.97 ± 0.78 at baseline and − 0.99 ± 0.88 prior to AIT initiation, while corresponding values for patients with ISS were − 2.15 ± 0.72 and − 1.04 ± 0.79, respectively. In patients evaluated after 2 years of concomitant AIT, mean HSDS had decreased to − 0.40 ± 1.16 and − 0.65 ± 0.52 for patients with GHD and ISS, respectively. Patients with GHD had a mean bone age/chronological age ratio (BA/CA) of 0.91 ± 0.11 at baseline and 0.97 ± 0.10 prior to AIT initiation, while corresponding values for patients with ISS were 0.85 ± 0.16 and 0.99 ± 0.10, respectively. In patients evaluated after 2 years of concomitant AIT, mean BA/CA values were 0.95 ± 0.10 and 0.96 ± 0.06 for patients with GHD and ISS, respectively.

**Conclusions:**

In this real-world analysis, use of AIT with GH in males appeared to be associated with ongoing growth over 2 years, and AIT likely augmented growth potential as indicated by continued HSDS increase with decreased BA/CA after AIT initiation.

**Trial registration:**

This trial was sponsored by Novo Nordisk and is registered with ClinicalTrials.gov (NCT01009905). Registered November 11, 2009; retrospectively registered

## Introduction

Idiopathic short stature (ISS) is a clinical condition defined as non-growth hormone (GH)-deficient short stature characterized by a height below 2 SD of the mean for age in the absence of any endocrine, metabolic, or other known condition that explains the short stature [1–3].

Recombinant GH was first introduced in 1985 and is effective in increasing short-term growth rates and attainment of adult height in patients with various growth disorders [4–9]. Recombinant GH was approved as a therapy in the United States in 2003 for children with ISS whose HSDS was ≥2.25 SD below the mean for age and sex who demonstrate growth rates unlikely to permit attainment of normal adult height [10]. Results from a meta-analysis suggested that GH therapy can increase final adult height by about 4 cm in patients with ISS; however, individual response is highly variable [10], and individual patient factors such as genetics, biochemical variability, and diagnosis can affect responsiveness to GH therapy [11].

The consensus statement by the European Society for Paediatric Endocrinology suggests treatment for patients with ISS using anabolic steroids, insulin-like growth factor 1 (IGF-1), or gonadotropin-releasing hormone after appropriate evaluation [1]. Aromatase inhibitor therapy (AIT) is also a suggested form of treatment, and functions by blocking the conversion of androgens to estrogens, thereby delaying epiphyseal fusion and bone age advancement [1, 12]. AIT has shown promising results for the treatment of boys with ISS and GH deficiency (GHD) when used as monotherapy or when combined with GH [13–16].

Norditropin® is a recombinant human GH indicated for several conditions associated with GHD; it is administered at 0.17 to 0.24 mg/kg/wk. for GHD and up to 0.47 mg/kg/wk. for ISS [17]. The non-interventional American Norditropin® Studies: Web-Enabled Research (ANSWER) Program initiated in 2002 was designed to assess real-life clinical outcomes of pediatric and adult patients treated with Norditropin® as prescribed by physicians according to standard clinical practice [18]. Data prospectively collected through ANSWER provided the opportunity to detail effects of treatment with GH in specific diagnostic and demographic populations [8, 19, 20]. The current study used data from ANSWER to assess height outcomes in children with ISS and GHD treated with combination GH and AIT.

## Methods

### Data source

Data were extracted from the prospective, non-interventional ANSWER program, which aimed to evaluate long-term safety and effectiveness outcomes of US pediatric and adult patients treated with Norditropin® (somatropin [rDNA origin] injection; Novo Nordisk A/S, Bagsværd, Denmark) [17]. The methodology and rationale for ANSWER have been described previously [18]. Briefly, ANSWER was a non-interventional initiative that enrolled adults and children previously naïve to GH treatment who were prescribed Norditropin® for treatment of GHD or other growth disorders. Patient histories and physical examination data were entered by participating physician investigators at their respective registry sites using the Web-based ANSWER program registry reporting form. GH doses administered to participants were determined by the treating physicians, and data collected at baseline included parameters such as baseline height standard deviation score (HSDS), weight, bone age (BA), maximal stimulated serum GH concentration, serum IGF-1 levels, and GH dose/frequency [18]. The ANSWER study collected patient data over a 14-year period (24 June 2002 to 30 September 2016), originally from 207 participating sites within the United States, of which 144 remained active by the end of the study. Of an initial 20,204 pediatric patients enrolled in ANSWER, 12,660 patients contributed to effectiveness outcomes assessments (GHD *n* = 8580; ISS *n* = 2367; other conditions *n* = 1713) [21].

### Assessments

For the current analysis, a longitudinal cohort approach was used to compare patient characteristics of GH-naïve, male patients diagnosed with GHD (defined as peak GH level of < 10 ng/mL [20 mIU/L] based on the local clinician’s choice of stimulation test) or ISS who had initiated treatment with GH and AIT during the observation period. A diagnosis of ISS was made at the discretion of the local health care provider, and was likely most often based on patient height being > 2 SD below normal for an unknown cause; however, other criteria such as the child’s height in relation to mid-parental height may have also been considered.

Chronologic age (CA), HSDS, BA, and the BA/CA ratio were analyzed at baseline, at the start of AIT, and after AIT initiation at local registry sites. BA was neither standardized nor blinded, and was determined based on provider and/or radiologist interpretation, and, along with BA/CA ratio data, were determined using a 6-month pre- and post-clinic visit window while HSDS data were determined using a 3-month pre- and post-clinic visit window.

At the start of AIT, patients were classified according to their Tanner stage, primarily based on reported genitalia development or pubic hair if data on genitalia were not available.

Mean and standard deviation for analyzed parameters were calculated; however, statistical comparisons were not made because of the limited number of patients and variations in the number of data points at each time point.

## Results

### Patient characteristics

Data were available for 142 male GH-naïve patients with GHD (*n* = 115) or ISS (*n* = 27) with mean (± SD) CAs of 12.10 ± 3.00 and 10.76 ± 3.07 years, respectively; and HSDS of − 1.97 ± 0.78 and − 2.15 ± 0.72, respectively (Table 1).

**Table 1:** Baseline demographic characteristics of patients

Mean ± SD	GHD (***n*** = 115)	ISS (***n*** = 27)
**Chronological age, y**	12.10 ± 3.00	10.76 ± 3.07
**Male, n (%)**	115 (100)	27 (100)
**HSDS**	−1.97 ± 0.78	−2.15 ± 0.72
**Target HSDS**	*n* = 96 − 0.36 ± 0.79	*n* = 24 − 0.80 ± 0.93
**IGF-1 SDS**	*n* = 66 − 1.15 ± 2.08	*n* = 17 − 0.65 ± 1.51
**Bone age, y**	*n* = 60 11.47 ± 2.76	*n* = 15 8.88 ± 3.11
**BA/CA ratio**	*n* = 60 0.91 ± 0.11	*n* = 15 0.85 ± 0.16
**Peak GH, ng/mL**	*n* = 50 6.25 ± 2.53	*n* = 5 20.04 ± 15.37
**BMI, kg/m**^**2**^	18.28 ± 3.17	17.83 ± 2.49
**BMI SDS**	−0.13 ± 1.13	0.09 ± 1.09
**Tanner stage, n (%)**		
**Not reported**	31 (27)	2 (7)
**I**	37 (32)	17 (63)
**II**	27 (23)	5 (19)
**III**	15 (13)	1 (4)
**IV**	4 (3)	1 (4)
**V**	1 (1)	1 (4)
**Total**	115 (100)	27 (100)

*BA* Bone age, *BMI* Body mass index, *CA* Chronologic age, *GH* Growth hormone, *GHD* Growth hormone deficiency, *HSDS* Height standard deviation score, *IGF-1* Insulin-like growth factor 1, *ISS* Idiopathic short stature, *SDS* Standard deviation score

As shown in Table 2, the mean CAs at initiation of AIT were 14.70 ± 1.85 and 13.76 ± 1.73 years for patients with GHD and ISS, respectively. Patients with GHD or ISS who had initiated GH therapy but had not yet initiated AIT showed increases in HSDS compared with baseline. Specifically, among patients with GHD, HSDS improved from a baseline value of − 1.97 ± 0.78 to − 0.99 ± 0.88 at AIT initiation, while patients with ISS showed an improvement from − 2.15 ± 0.72 at baseline to − 1.04 ± 0.79 at AIT initiation. Patients also showed advancing BA during the GH treatment period prior to AIT initiation. Among patients with GHD, BA increased from 11.47 ± 2.76 years at baseline to 13.54 ± 2.41 years at AIT initiation, while the corresponding values for patients with ISS were 8.88 ± 3.11 years and 13.45 ± 0.97, respectively. Across the combined GHD and ISS patient population, 7 (5%) patients were classified at Tanner stage I, while 95 (65%) were classified at Tanner stages II to V (the remaining 29% did not have available Tanner stage data) (not shown).

**Table 2:** Patient characteristics at AIT initiation

Mean ± SD	GHD	ISS
**Chronological age, y**	*n* = 115 14.70 ± 1.85	*n* = 27 13.76 ± 1.73
**HSDS**	*n* = 112 −0.99 ± 0.88	*n* = 27 −1.04 ± 0.79
**Bone age, y**	*n* = 91 13.54 ± 2.41	*n* = 23 13.45 ± 0.97
**BA/CA ratio**	*n* = 90 0.97 ± 0.10	*n* = 23 0.99 ± 0.10

*AIT* Aromatase inhibitor therapy, *BA* Bone age, *CA* Chronologic age, *GHD* Growth hormone deficiency, *HSDS* Height standard deviation score, *ISS* Idiopathic short stature

### HSDS

A longitudinal analysis was performed to investigate changes in HSDS from baseline to 1 year after AIT initiation. In patients with GHD and data available from baseline through 1 year of AIT (*n* = 72), the mean HSDS improved from − 1.94 ± 0.77 at baseline to − 0.92 ± 0.89 at AIT initiation, and then further improved to − 0.62 ± 0.95 after 1 year of concomitant GH plus AIT (Table 3). Corresponding values for patients with ISS (*n* = 19) were − 2.07 ± 0.77 at baseline, − 0.87 ± 0.86 at AIT initiation, and − 0.69 ± 0.83 after 1 year of concomitant GH plus AIT (Table 3). Among patients with GHD and data available 2 years after AIT initiation (*n* = 27), mean HSDS improved over time from − 2.21 ± 0.93 at baseline to − 1.00 ± 0.96 at AIT initiation, and then further to − 0.40 ± 1.16 after 2 years of concomitant GH plus AIT (Table 4). Corresponding values for patients with ISS and available data (*n* = 9) were − 2.44 ± 0.27 at baseline, − 0.85 ± 0.85 at AIT initiation, and − 0.65 ± 0.52 after 2 years of concomitant therapy (Table 4).

**Table 3:** Longitudinal data for patients with GHD or ISS with growth disorders and 1-year data

Mean ± SD		GHD	ISS
**HSDS**	**Baseline**	*n* = 72 − 1.94 ± 0.77	*n* = 19 −2.07 ± 0.77
	**Start of AIT**	*n* = 70 − 0.92 ± 0.89	*n* = 19 − 0.87 ± 0.86
	**1 year after AIT**	*n* = 72 − 0.62 ± 0.95	*n* = 19 −0.69 ± 0.83
**BA/CA ratio**	**Baseline**	*n* = 36 0.90 ± 0.11	*n* = 9 0.86 ± 0.19
	**Start of AIT**	*n* = 56 0.96 ± 0.10	*n* = 17 0.99 ± 0.11
	**1 year after AIT**	*n* = 43 0.93 ± 0.09	*n* = 16 0.96 ± 0.08

*AIT* Aromatase inhibitor therapy, *BA* Bone age, *CA* Chronologic age, *GHD* Growth hormone deficiency, *HSDS* Height standard deviation score, *ISS* Idiopathic short stature

**Table 4:** Longitudinal data for patients with GHD or ISS with growth disorders and 2-year data

Mean ± SD		GHD	ISS
**HSDS**	**Baseline**	*n* = 27 − 2.21 ± 0.93	*n* = 9 −2.44 ± 0.27
	**Start of AIT**	*n* = 25 −1.00 ± 0.96	*n* = 9 − 0.85 ± 0.85
	**2 years after AIT**	*n* = 27 − 0.40 ± 1.16	*n* = 9 −0.65 ± 0.52
**BA/CA ratio**	**Baseline**	*n* = 16 0.90 ± 0.11	*n* = 5 0.79 ± 0.20
	**Start of AIT**	*n* = 23 0.98 ± 0.12	*n* = 9 1.02 ± 0.11
	**2 years after AIT**	*n* = 10 0.95 ± 0.10	*n* = 8 0.96 ± 0.06

*AIT* Aromatase inhibitor therapy, *BA* Bone age, *CA* Chronologic age, *GHD* Growth hormone deficiency, *HSDS* height standard deviation score; ISS, idiopathic short stature

### BA/CA ratio

The longitudinal analysis also investigated changes in BA/CA ratios over time. Overall, the results showed accelerated increases in BA/CA before AIT but decreases after AIT. For patients with GHD and available data 1 year after AIT initiation, the mean BA/CA ratio increased from 0.90 ± 0.11 (*n* = 36) at baseline to 0.96 ± 0.10 (*n* = 56) at AIT initiation, and then decreased slightly to 0.93 ± 0.09 (*n* = 43) after 1 year of AIT (Table 3). Corresponding values for patients with ISS were 0.86 ± 0.19 (*n* = 9) at baseline, 0.99 ± 0.11 (*n* = 17) at AIT initiation, and 0.96 ± 0.08 (*n* = 16) after 1 year of AIT (Table 3). Similar outcomes were observed for patients with GHD or ISS and available data after 2 years of AIT. For patients with GHD, BA/CA ratio increased from 0.90 ± 0.11 (*n* = 16) at baseline to 0.98 ± 0.12 (*n* = 23) at AIT initiation, and 0.95 ± 0.10 (*n* = 10) after 2 years of AIT (Table 4). Corresponding values for patients with ISS were 0.79 ± 0.20 (*n* = 5) at baseline, 1.02 ± 0.11 (*n* = 9) at AIT initiation, and 0.96 ± 0.06 (*n* = 8) after 2 years of AIT (Table 4).

## Discussion

Results of this real-world analysis showed that use of AIT with GH in males appeared to be associated with ongoing growth over 2 years. Overall, BA/CA ratios showed accelerated increases before AIT, then decreases after AIT, whereas HSDS increased continuously. These findings are consistent with the hypothesis that AIT augments growth potential by decreasing estrogen synthesis and thereby delaying epiphyseal fusion, resulting in continued progression of HSDS while delaying BA advancement [22, 23].

For patients with GHD and ISS, AIT may be initiated during GH treatment in patients of advanced CA and Tanner stage and advancing BA based on physician concerns about a diminishing treatment window for optimizing growth during puberty due to skeletal maturation and impending epiphyseal fusion. Early studies on the pediatric use of the AI letrozole demonstrated that the closure of epiphyseal plates could be delayed in male adolescents with pubertal delay who were given testosterone to accelerate sexual maturation [15, 24, 25]. An observational study investigated whether recombinant GH (0.076 mg/kg*d) and anastrozole could increase height in sexually mature adolescents with ISS (baseline mean age, 15.2 years; BA, 14.5 years) who had almost reached their adult height but had yet to experience complete growth plate fusion. Both GH and GH plus anastrozole increased final height compared with historical controls, but the increase was significantly greater among patients who received the concomitant anastrozole. Compared with GH alone, significant improvements in growth velocity for GH plus anastrozole were observed through the second year of administration [26]. A study in slightly younger patients with ISS (baseline mean age, 14.0 years; BA, 12.7 years) revealed significant improvements in height gain after 3 years when anastrozole or letrozole were added to GH treatment vs GH alone [13].

The results from the current study are consistent with the previously observed effect of AIT in slowing bone maturation and prolonging the period during which GH treatment may increase growth potential [13, 14, 26]. Despite the observational nature of the current study and lack of statistical comparisons, these data are valuable in that they add to the previous body of work from clinical trials by demonstrating that increases in height can be achieved when AIT is added to GH in real-world conditions.

This study was limited by the use of retrospective data, and from very large patient registries. Data were collected by hundreds of practitioners from thousands of patients with diverse backgrounds in many countries, introducing potential variability at multiple levels. Bone ages were entered at each registry site based on provider and/or radiologist interpretation; therefore, results may have been entered by providers not blinded to AIT status. Additionally, patients were classified as ISS based on diagnoses assigned by their individual providers, which may have included varying degrees of clinical judgement along with guideline recommended criteria, and/or short stature may have occurred from causes unknown at the time of diagnosis, such as constitutional delay or genetic short stature. We were also limited by the use of real-world data collected during ongoing care of children in many different clinical settings, resulting in inconsistent patient numbers for different variables within each analysis. Considering, however, that we were starting with a limited number of patients, especially because of the rarity of the ISS diagnosis, we believe that all available data are of value and chose to include any/all patients with data available at each time point. Finally, our patient population consisted of young patients who were still actively growing, which yielded intermediate outcomes but not data on final adult height, which is the primary goal of treatment with GH and AIT for patients with these GHD, ISS, and other conditions where these therapies may be beneficial.

## Conclusion

In this real-world analysis of male patients with GHD or ISS, use of AIT with GH appeared to be associated with ongoing growth over 2 years. BA/CA ratios showed accelerated increases before AIT initiation, followed by decreases after AIT, whereas HSDS increased continuously. These findings suggest AIT augments growth potential by decreasing estrogen synthesis and thereby delaying epiphyseal fusion.

## Data Availability

The data that support the findings of this study are available from Novo Nordisk, but restrictions apply to the availability of these data, which were used under license for the current study, and so are not publicly available. Data are however available from the authors upon reasonable request and with permission of Novo Nordisk, Inc.

## Abbreviations

AIT: Aromatase inhibitor therapy
ANSWER: American Norditropin® Studies: Web-Enabled Research (Program)
BA: Bone age
CA: Chronologic age
GH: Growth hormone
GHD: Growth hormone deficiency
HSDS: Height standard deviation score
IGF-1: Insulin-like growth factor 1
ISS: Idiopathic short stature
US: United States
